# Ritodrine-Induced Agranulocytosis: A Case Report and Literature Review

**DOI:** 10.1155/2020/5846161

**Published:** 2020-08-08

**Authors:** Atsushi Daimon, Misa Nunode, Takumi Sano, Tomohito Tanaka, Daisuke Fujita, Masahide Ohmichi

**Affiliations:** Department of Obstetrics and Gynecology, Osaka Medical College, Osaka, Japan

## Abstract

Ritodrine hydrochloride is used for preterm labor, although serious side effects, including agranulocytosis, are reported. We report a case of ritodrine hydrochloride-induced agranulocytosis accompanied by bacteremia due to catheter infection. At 24 weeks of gestation, a female patient presented due to threatened premature labor and was administered continuous intravenous infusion of ritodrine hydrochloride. On day 36 after starting intravenous ritodrine hydrochloride, she was diagnosed with agranulocytosis. The white blood cell and granulocyte count nadirs were 1,660/*μ*l and 438/*μ*l. The cumulative dose of ritodrine hydrochloride was 2,610 mg. Ritodrine therapy was immediately stopped, and she was given an intravenous injection of antibiotics and granulocyte colony-stimulating factor. From her blood culture, methicillin-sensitive *Staphylococcus aureus* was detected. However, she started vaginal delivery two days after we stopped the ritodrine infusion. When using ritodrine hydrochloride, it is necessary to frequently check the white blood cell count, regardless of the total dose and treatment period.

## 1. Introduction

Ritodrine, a beta-2 adrenergic agonist, is a widely used tocolytic agent for treating preterm uterine contractions. However, it has some maternal side effects, such as tachycardia, elevated liver enzymes, pulmonary edema, impaired glucose tolerance, finger shivering, and, rarely, rhabdomyolysis and agranulocytosis [1, 2].

We, herein, report a case of agranulocytosis due to continuous infusion therapy with ritodrine and summarize ritodrine-induced agranulocytosis.

## 2. Case Presentation

**Figure 1:**
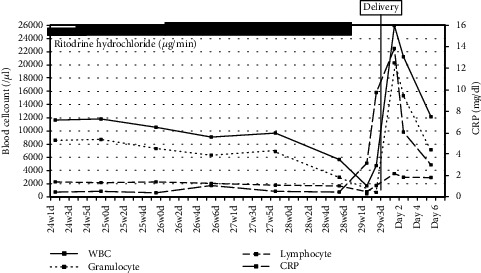
Changes in the WBC, granulocyte, lymphocyte, and CRP values from 24 weeks of gestation to 6 days postpartum.

A 38-year-old woman (gravida 1, para 0) was admitted with complaints of uterine contractions at 24 weeks and 1 day of gestation. At admission, her white blood cell (WBC) count was 11,600/*μ*l, with 73.7% segmented neutrocytes. We started continuous intravenous ritodrine hydrochloride treatment at a dose of 50 *μ*g/min, and the dose was increased to 75 *μ*g/min according to uterine contractions and the cervical length (Figure 1). Routine blood tests were performed once a week. On day 36 after the start of therapy, she developed a fever of 39°C, and redness and swelling were found at the intravenous drip penetration point. We suspected an intravascular indwelling catheter infection, and blood tests and cultures were performed. Her WBC count was 1,660/*μ*l, with 26.4% segmented neutrocytes (438/*μ*l), and the C-reactive protein (CRP) level was elevated to 3.15 mg/dl (Table 1). Drug-induced agranulocytosis by ritodrine was diagnosed. Ritodrine therapy (cumulative dose of 2,610 mg) was immediately stopped. We started an intravenous injection of an antibiotic (cefazolin (CEZ) 2 g/day) and intramuscular injection of 75 *μ*g/body of granulocyte colony-stimulating factor (G-CSF). On day 37 after the start of therapy, gram-positive cocci suspicious for *Staphylococci* were detected in her blood culture. Considering the possibility of methicillin-resistant *Staphylococcus aureus* (MRSA), the antibiotic was changed to vancomycin hydrochloride (2 g/day). On day 38 after the start of therapy, the patient developed frequent uterine contractions and started magnesium sulfate (1 g/h) administration. However, her uterine contractions strengthened, resulting in the vaginal delivery of a 1,288 g male infant with an Apgar score of 7 at 1 min and 8 at 5 min. On day 2 after delivery, her blood culture result revealed methicillin-sensitive *Staphylococcus aureus* (MSSA); therefore, her antibiotics were switched to CEZ (3 g/day). On day 5 after delivery, her WBC count rose to 12,120/*μ*l, with 59.0% segmented neutrocytes (7,151/*μ*l), and her CRP decreased to 2.99 mg/dl. She was discharged without infectious complications. Her infant was placed in the neonatal intensive care unit, had an uncomplicated neonatal course, and was discharged without any morbidity on day 71 after delivery.

## 3. Discussion

Ritodrine hydrochloride is the most frequently used and most effective tocolytic agent for patients with preterm labor in Japan. The side effects of ritodrine hydrochloride include tachycardia, elevated liver enzymes, pulmonary edema, impaired glucose tolerance, finger shivering, and, rarely, rhabdomyolysis and agranulocytosis. Ritodrine hydrochloride-induced agranulocytosis was first reported in 1986 [1]. We searched for case reports or clinical studies on ritodrine-induced agranulocytosis in the PubMed database and the database of the Japan Medical Abstracts Society (1986–2017) and found 40 studies and 47 case reports of the disorder, including our own; 44 cases were reported in Japan, and 1 case each was reported in the USA, China, and Taiwan. The mean duration of ritodrine therapy was 30.3 days (range, 21–69 days), and the mean cumulative ritodrine dose based on 38 cases for which the necessary data were reported was 6,642 mg (range, 2,000–17,700 mg) (Table 2). This result was similar to that of a previous report [3]. All patients stopped receiving ritodrine after being diagnosed with ritodrine-induced agranulocytosis. Thirty-five patients (74.5%) were treated with G-CSF, and 31 patients (66.0%) were administered antibiotics. One patient was diagnosed with a pulmonary *Aspergillus* infection, which was treated with oral itraconazole [4]. All patients were discharged without any complications. The preterm birth rate was 53.2% of the patients who developed ritodrine-induced agranulocytosis. In previous reports, there were no cases with positive blood cultures [5], and this case was the first in which causative bacteria (MSSA) were detected in the blood culture test. As inflammatory cytokines, such as IL-6 and TNF-*α*, are known to be increased before and after parturition in cases of premature birth [6], the bacteremia-induced elevation of inflammatory cytokines may have caused preterm birth in our patient.

As no specific symptoms of agranulocytosis have been described, it is detected in many cases by regular blood tests. When using ritodrine hydrochloride, physicians should regularly check the WBC count, regardless of the total dose and treatment period. The lower normal cutoff values of WBCs and granulocytes during pregnancy are 6,000/*μ*l and 3,800/*μ*l, respectively [7]. When values drop below these numbers, the onset of agranulocytosis should be suspected and more frequent blood tests should be considered.

## Figures and Tables

**Table 1:** Blood examination findings at the onset of agranulocytosis.

WBC (/*μ*l)	1,660	TP (g/dl)	6.3
RBC (10^3^/*μ*l)	3,760	Alb (g/dl)	3.2
Hb (g/dl)	11.9	T-Bil (mg/dl)	0.3
Plt (10^3^/*μ*l)	368	AST (U/L)	22
Granulocyte (/*μ*l)	438	ALT (U/L)	25
Lymphocyte (/*μ*l)	774	BUN (mg/dl)	5
CRP (mg/dl)	3.15	Cre (mg/dl)	0.44

**Table 2:** The 47 cases found in our literature review of ritodrine hydrochloride-induced agranulocytosis in pregnancy.

Mean maternal age (range) (years)	29.6 (21-40)
Multiparous (%)	15 (31.9)
Multiple pregnancy (%)	14 (29.8)
Ritodrine therapy	
Mean duration (range) (days)	30.3 (21-69)
Mean cumulative dose (range) (mg)	6,642 (2,000-17,700)
Labor date at onset of agranulocytosis	
WBC (range) (/*μ*l)	1,864 (790-3,500)
Granulocyte (range) (/*μ*l)	216 (0-1,925)
Concomitant drug	
Magnesium sulfate (%)	17 (36.2)
Indomethacin (%)	5 (10.6)
Infection signs (%)	21 (44.7)
Antibacterial (%)	31 (66.0)
G-CSF (%)	35 (74.5)
Preterm birth (%)	25 (53.2)

## Abbreviation

WBC: White blood count
CRP: C-reactive protein
CEZ: Cefazolin
G-CSF: Granulocyte colony-stimulating factor
MRSA: Methicillin-resistant *Staphylococcus aureus*
MSSA: Methicillin-sensitive *Staphylococcus aureus.*
